# Therapeutic Effects of Kangzhi Syrup in a Guinea Pig Model of Ovalbumin-Induced Cough Variant Asthma

**DOI:** 10.1155/2018/5867835

**Published:** 2018-05-23

**Authors:** Youpeng Wang, Pengyu Zhu, Jiejun Tan, Zhiwei Liu, Wanying Qu, Lujia Liu, Zhijun Li

**Affiliations:** ^1^The Second Affiliated Hospital of Heilongjiang University of Traditional Chinese Medicine, Harbin, Heilongjiang 150001, China; ^2^The Third People's Hospital of Bengbu, Bengbu, Anhui 233000, China; ^3^Heilongjiang University of Traditional Chinese Medicine, Harbin, Heilongjiang 150040, China

## Abstract

**Purpose:**

This study aimed to investigate the possible effects and underlying mechanisms of Kangzhi syrup on ovalbumin- (OVA-) induced cough variant asthma (CVA) in guinea pigs.

**Methods:**

All 48 guinea pigs were randomly assigned to four experimental groups: normal, OVA model with or without Kangzhi syrup (OVA and OVA + KZ), and OVA with Dexamethasone (OVA + DM). After sensitizing the guinea pigs, a cough challenge was performed by the inhalation of capsaicin. The antitussive effect, inflammatory cells, cytokines in bronchoalveolar lavage fluid (BALF) and lung tissue, and morphological changes were examined.

**Results:**

Compared with model group, Kangzhi syrup effectively exerted an antitussive effect (*p* < 0.0001) and reduced the pneumonic anaphylacticitis by inhibiting the infiltration of total inflammatory cells (*p* < 0.0001) and reducing the percentage of eosinophil in BALF (*p* < 0.0001). Moreover, evidence from morphological studies also demonstrated that Kangzhi syrup inhibited the infiltration of inflammatory cells and ameliorated the structure changes. NF-*κ*B and TGF-*β*1 expression were attenuated in the OVA + KZ group versus the OVA group (*p* < 0.0001). Additionally, a semiquantitative analysis of TGF-*β*1 expression also demonstrated that the Kangzhi syrup attenuated this profibrogenic growth factor (*p* < 0.001).

**Conclusions:**

The results demonstrated that Kangzhi syrup exerted a considerable antitussive effect in CVA animal model, which depended on its marked impact on the anti-anaphylactic inflammation. Additionally, it could ameliorate the airway remodeling by inhibiting NF-*κ*B and TGF-*β*1 signal pathway.

## 1. Introduction

The chronic cough is a common and frustrating symptom of respiratory disease. One of the most representative airway disorders responsible for a chronic cough is asthma, which is characterized by airway hyperresponsiveness (AHR), chronic inflammation, tissue remodeling, and mucus hypersecretion [1] and can be subclassified into an atopic cough, eosinophilic bronchitis, and cough variant asthma (CVA) [2]. The main symptom of CVA is prolonged nonproductive cough, accompanied by increased cough sensitivity and increased bronchial responsiveness, and eosinophilia infiltration of central and peripheral airways [3]. In general, CVA is regarded as a precursor of asthma, and about 35.7% of patients with CVA will become classic asthma in 5 years [4]. A multicenter survey on causes of the chronic cough demonstrated that CVA accounted for 32.6% of the total patients with chronic cough across five regions in China [5]. Thus, CVA is one of the major causes of chronic cough and impacted seriously on the patient's quality of life.

The underlying mechanism of pathogenesis for CVA is still elusive. A progress in the pathobiology of asthma pointed out that the type 2 immune response was associated with atopic diseases, such as allergy and asthma, mainly mediated by eosinophil, T helper 2 cells, and IgE-producing B cells [6]. It is generally accepted that the persistent inflammation results in hypersensitivity to allergens and remodeling of the epithelium and subepithelial fibrosis. The glucocorticoids have long been the optimal therapeutic regimen to control asthma by suppressing the inflammation. As glucocorticoids have multiple side effects, particularly in prolonged high doses, asthma or CVA often starts in childhood and becomes persistent for years. A long-term glucocorticoids usage not only causes serious side effects, but also influences the development of the immune system in early childhood. Thus, a complementary or alternative medicine (CAM) has been a hotspot of research. Herbal remedies are now one of the most popular forms of CAM to treat asthma; nearly 40% of people with asthma once used herbal remedies [7]. There were studies that support the efficacy of herbal remedies in improving lung function, reducing the use of glucocorticoids [8, 9]. In a noncomparative, multicenter trial, an extract from* Magnoliae Flos* was applied as an adjuvant besides inhaled corticosteroid therapy and showed beneficial effects on both lung functions and asthma symptoms [10]. Moreover, a plant-based remedy is often perceived as being “natural” and “safe”; it is consistently accepted and preferred by patients.

Herbal remedies have been used for centuries to treat the diseases of respiratory system. In addition to traditional Chinese formulae, several asthma drugs were derived from herbal medicine. For example, theophylline was extracted from tea leaves, and “Mahuang” was the origin of ephedrine. Kangzhi syrup was developed from the* Belamcanda* and* Ephedra* Decoction, a traditional Chinese formula, composed of 15 traditional Chinese herbs. It has an obvious effectiveness and safety in treating chronic cough and CVA with the low recurrence rate after medication discontinuation and has been used for decades in the Second Affiliated Hospital of Heilongjiang University of Chinese Medicine. The possible mechanism of Kangzhi syrup in asthma therapy has not yet been thoroughly investigated, even though some herbs in this formula were proven to have potential efficacy in controlling inflammatory diseases [11–13]. Therefore, the present study utilized a guinea pig model for CVA and attempted to investigate the possible underlying molecular mechanisms of Kangzhi syrup [14].

## 2. Materials and Methods

### 2.1. Preparation of Kangzhi Syrup

Kangzhi syrup is a commercial preparation processed by the Preparation Center of Second Affiliated Hospital of Heilongjiang University of Chinese Medicine (Harbin, China). It is comprised of 15 traditional Chinese medicines:* Ephedrae Herba* (Mahuang);* Gypsum Fibrosum* (Shigao);* Belamcandae Rhizoma* (Shegan);* Armeniacae Semen Amarum* (Kuxingren);* Stemonae Radix* (Baibu);* Mori Cortex* (Sangbaipi);* Fritillariae Ussuriensis Bulbus* (Pingbeimu);* Scutellariae Radix* (Huangcen);* Houttuyniae Herba* (Yuxingcao);* Polygoni Cuspidati Rhizoma Et Radix* (Huzhang);* Bistortae Rhizoma *(Quanshen);* Phragmitis Rhizoma* (Lugen);* Bombyx Batryticatus* (Jiangcan);* Pheretima* (Dilong);* Glycyrrhizae Radix Et Rhizoma* (Gancao). According to the dose of clinical application per body surface area, Kangzhi syrup (10 ml/ampoule, equals to 10.95 g crude medicinal materials) was converted to an equivalent dose for adult guinea pig at 5 g/kg and was made to be 0.5 g/100 ml solution with sterile water before treating animals.

### 2.2. Reagents

Kangzhi syrup and Dexamethasone (DM) were obtained from the Second Affiliated Hospital of Heilongjiang University of Chinese Medicine. Ovalbumin (OVA) and methacholine (Sigma Aldrich, Co., St Louis, USA), Imject Alum Adjuvant (Thermo Scientific, Rockford, IL, USA), and Wright-Giemsa staining (Baso Diagnostic Inc., Zhuhai, China) were well prepared for the research. Total RNAs isolation kits and RT Reagent Kit were purchased from BioTeke Corporation (BioTeke, China). Anti-NF-*κ*B antibody for Western blotting was purchased from Abcam. Anti-TGF-*β*1 antibody for Western blotting was purchased from Biorbyt. All kits for enzyme-linked immunosorbent assay were purchased from R&D system.

### 2.3. Experimental Animals

A total of 48 male guinea pigs (6–8 weeks, 200 ± 30 g) were obtained from the Experimental Animal Center of Second Affiliated Hospital of Heilongjiang University of Chinese Medicine, which were maintained in an animal facility under standard laboratory conditions for 1 week prior to experiments. All animal procedures were strictly conducted in accordance with the international ethical guidelines and National Institutes of Health guide concerning the Care and Use of Laboratory Animals and were approved by the Animal Care and Use Committee of Heilongjiang University of Chinese Medicine.

### 2.4. The Score Criterion of Ashcroft Grading Scale

Based on the published article [15], the H&E stained histological sections were used to analyze by visual assessment. The characterization of Ashcroft scale is shown in Table 1.

### 2.5. Sensitization and OVA Challenge

**Figure 1:**
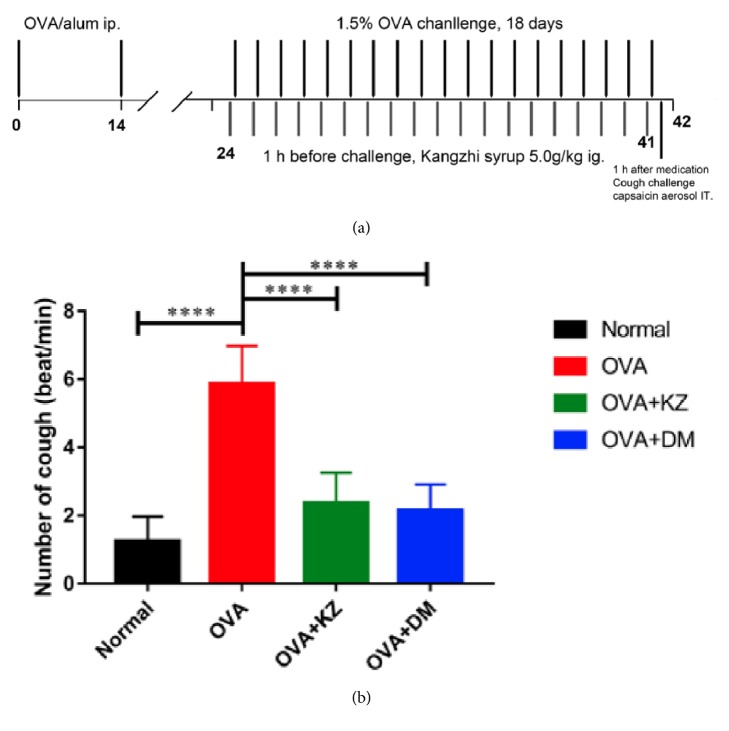
Effect of Kangzhi syrup on the cough induced by the inhalation of capsaicin aerosol in OVA-sensitized guinea pigs. (a) Time line representation of the CVA model, pharmacological intervention, and cough challenge. (b) Kangzhi syrup decreased the number of coughs induced by the inhalation of capsaicin aerosol in OVA-sensitized guinea pigs. Data are mean ± SEM; ^*∗∗∗∗*^*p* < 0.0001.

All guinea pigs were randomly separated into 4 groups as follows (*n* = 12): normal group, OVA group, OVA + KZ groups (5 g/kg), and OVA + DM group (2.5 mg/kg). The procedures of sensitization, challenge, and treatment protocols are slightly modified from the report and are schematized in Figure 1 [16]. Guinea pigs for CVA were sensitized on day 0 and day 14 by intraperitoneal (i.p.) injection of 4% OVA in 0.5 ml and 0.2 ml of 2% aluminum hydroxide. 10 days after the second sensitization, the guinea pigs were challenged with 1.5% OVA through aerosol inhalation for 45 min for 18 days, and the normal group with saline instead. The criterions about the successful CVA model are the obvious eosinophilia infiltration in the lung tissue, the airway hypersensitivity, and the nonproductive cough triggered by inhalation of capsaicin aerosol. 1 hour before each challenge, OVA + KZ group was treated through intragastric administration (i.g.) and OVA + DM group accepted Dexamethasone 0.5 mg/kg (i.g.).

### 2.6. Cough Measurement

Each guinea pig was placed in a transparent sealed chamber and exposed to capsaicin aerosol for cough challenges 60 min after the last drug treatment. The mixed airflow of capsaicin aerosol (10^−4^ mol/L) into the chamber was provided at 600 ml/min through one end and out the opposite end. Two minutes later, the mixed airflow of capsaicin aerosol in the chamber was neutralized with fresh air, and then the number of coughs was recorded over the following 2 min. The cough sounds were amplified by a wireless microphone placed in the box and recognized from the characteristic posture (splaying of the front feet and forward stretching of the neck and opening of the mouth). The number of coughs was recorded and analyzed by a trained observer.

### 2.7. Cellular Staining of BALF

The collection of BALF (bronchoalveolar lavage fluid) was performed 24 h after the last drug treatment. The animals were sacrificed and the trachea was exposed and cannulated with a 20 G needle. The left lungs were removed from the distal of the ligature near the bifurcation for the further histological assessment. The right lungs were lavaged with three successive ice cold saline doses (4 ml) via cannula to collect 10 ml of BALF, and then cell counting was performed. After cell counting, BALF was centrifuged at 1500 rpm, 4°C, for 10 min to obtain inflammatory cells. BALF supernatant was frozen at −80°C for cytokines analysis. The right lungs were frozen in the liquid nitrogen and preserved at −80°C for extraction of protein and RNA.

### 2.8. ELISA Assay of Cytokines

BALF in each group mentioned above were used for quantitative assay of IL-1*β*, INF-*γ*, and TGF-*β*1 by R&D systems Quantikine Colorimetric Sandwich ELISA Kits according to the manufacturer's instructions. The serum level of eosinophil cationic protein (ECP), reflecting ongoing eosinophilic inflammation of the airways, was measured by R&D systems Quantikine Colorimetric Sandwich ELISA Kits.

### 2.9. Histological Evaluation and IHC Staining

The left lungs were fixed in 10% neutral formalin overnight. The fixed tissues were cut into 5 × 5 mm small pieces, embedded in paraffin, which was performed on 5 *μ*m sections, and stained following standard procedures of H&E and IHC staining. The TGF-*β*1 was detected with specific rabbit polyclonal antibody using streptavidin-biotin immunoenzymatic antigen detection system. The pictures were acquired using the OLYMPUS BX60 microscope at an original magnification of ×400 and analyzed by an image-processing system of Motic Med 6.0. The H&E staining sections were scored according to the modified Ashcroft grading scale [15].

### 2.10. Quantitative Real-Time PCR

Total RNA samples from lungs after stimulation were extracted using RNApure total RNA fast isolation kit according to manufactures' protocol. 1 *μ*g RNA was reverse-transcribed to cDNA in 20 *μ*l using cDNA synthesis kit. Quantitative real-time PCR was performed using SYBR Green Master Mix with Bioneer detection system. The specific primer sequences for RNA amplification were as described as follows:

 
*β*-actin Forward: 5′-GGCTGTATTCCCC-TCCATCG-3′,
 
*β*-actin Reverse: 5′-CCAGTTGGTAACAATGCCATGT-3′;
  TGF-*β*1 Forward: 5′-CCAGAGTGGTTGTCCTTTGATG -3′,
  TGF-*β*1 Reverse: 5′-GACCGATCCCGTTGATTTCC -3′;
  NF-*κ*B Forward: 5′-GATGATCGTCACCGGATTGAG -3′,
  NF-*κ*B Reverse: 5′-ACTGAGGGATGGTGGAAAGGT -3′.

### 2.11. Western Blotting

Lung tissue samples were homogenized in lysis buffer containing proteinase and phosphatase inhibitor cocktail. NF-*κ*B and TGF-*β*1 were detected with specific antibodies, and *β*-actin levels were used as an internal control.

### 2.12. Statistical Analysis

All data are expressed as mean ± standard error of the mean (SEM). One-way analysis of variance (ANOVA) was used to analyze the statistical differences among the groups. The *p* values < 0.05 were considered statistically significant. Statistical analyses and graphs were performed with SPSS 15.0 for Windows and GraphPad Prism 7.0 software.

## 3. Results

### 3.1. Measurement of the Cough Numbers Induced with Aerosolized Capsaicin In Vivo

The results revealed that the number of coughs induced by aerosolized capsaicin was significantly increased by 5-fold in the OVA group when compared with normal saline (*p* < 0.0001). Kangzhi syrup treatment significantly decreased the number of coughs, as effective as the treatments of the Dexamethasone (OVA versus OVA + KZ, *p* < 0.0001; OVA versus OVA + DM, *p* < 0.0001) (Figure 1(b)).

### 3.2. The Number of the Inflammatory Cells in BAL Fluid

**Figure 2:**
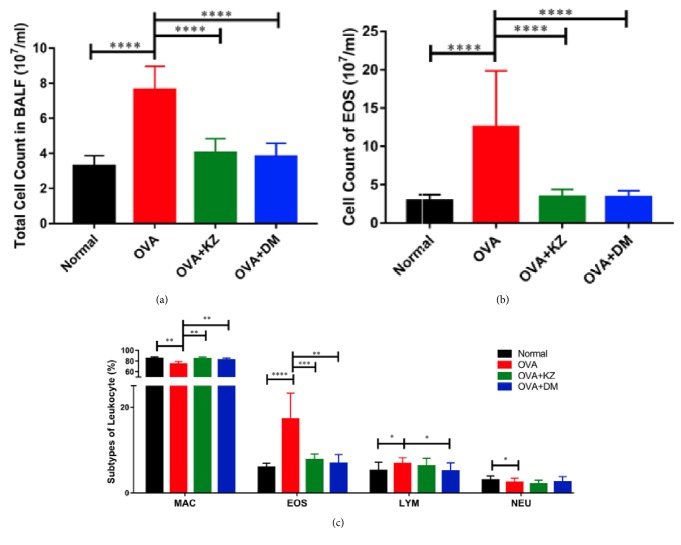
Effects of Kangzhi syrup on the inflammatory cells infiltration in BALF. (a) Compared with OVA group, Kangzhi syrup and Dexamethasone significantly reduced the infiltration of total cells in BALF; (b) Kangzhi syrup and Dexamethasone significantly reduced the absolute number of EOS in BALF; (c) Kangzhi syrup and Dexamethasone reversed the imbalance of MAC (macrophage) and EOS (eosinophils) in the percentage of inflammatory cells. Data are mean ± SEM; ^*∗*^*p* < 0.05; ^*∗∗*^*p* < 0.01; ^*∗∗∗*^*p* < 0.001; ^*∗∗∗∗*^*p* < 0.0001.

Compared with the normal group, stimulation with 1.5% OVA markedly increased the total number of leukocytes in BALF by 2-fold (*p* < 0.01), along with the increase of eosinophil in the absolute number and the percentage (*p* < 0.0001). In addition, the percentage of lymphocytes also increased in the OVA group (*p* < 0.05), whereas the percentage of macrophages and neutrophils decreased in comparison to the normal group (*p* < 0.01 and *p* < 0.05, respectively). Kangzhi syrup inhibited the increase in the total number of leukocytes (*p* < 0.0001) and the percentage of eosinophil (*p* < 0.001), which indicated a significant inhibition of OVA induced inflammation. Likewise, the treatment of Dexamethasone also exerted the similar effects with Kangzhi syrup on the leukocytes and eosinophil (*p* < 0.0001 and *p* < 0.01, respectively). The percentage of lymphocytes was not affected by the treatments of Kangzhi syrup and inhibited by the Dexamethasone (*p* < 0.05, Figure 2).

### 3.3. Concentration Determination of IL-1*β*, IFN-*γ*, TGF-*β*1, and ECP in the BALF

**Figure 3:**
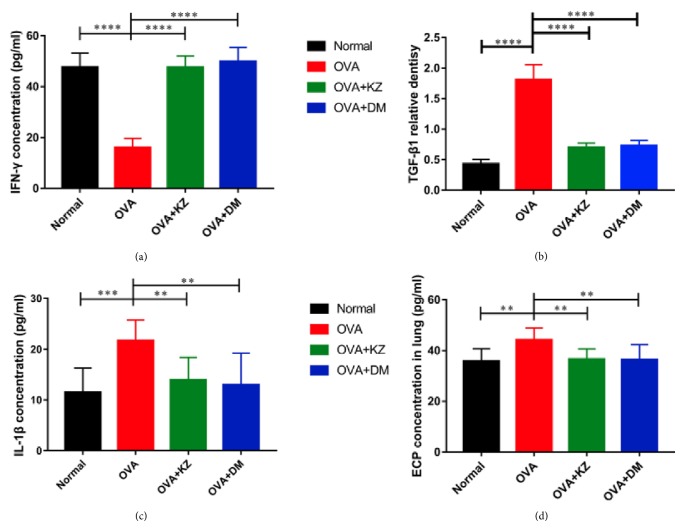
Effects of Kangzhi syrup on the concentration of proinflammatory cytokines in the BALF. (a–c) The concentrations of INF-*γ*, IL-1*β*, and TGF-*β*1 in the BALF were determined by ELISA kits. Kangzhi syrup exerted the similar effects with Dexamethasone in decreasing the concentrations of IL-1*β* and TGF-*β*1 and reversed the concentration of INF-*γ*. (d) The concentrations of eosinophil cationic protein (ECP) in serum were measured by ELISA kits; the concentration of ECP was decreased by the treatment of Kangzhi syrup and Dexamethasone. Data are mean ± SEM; ^*∗∗*^*p* < 0.01; ^*∗∗∗∗*^*p* < 0.0001.

We next examined the effect of Kangzhi syrup on the inflammatory cytokines such as IL-1*β*, IFN-*γ*, and TGF-*β* in the BALF. In the BALF, IL-1*β* and IFN-*γ* were increased by OVA challenge, and they were significantly decreased in Kangzhi syrup and Dexamethasone treated groups (Figure 3). TGF-*β*1 level was also significantly decreased by Kangzhi syrup and Dexamethasone and increased by OVA challenge (Figure 3). In addition, IFN-*γ* was suppressed by OVA challenge in the BALF and returned to the normal level by the treatments of Kangzhi syrup and Dexamethasone.

### 3.4. Exploration of Pathological Changes in the Lung Tissue

**Figure 4:**
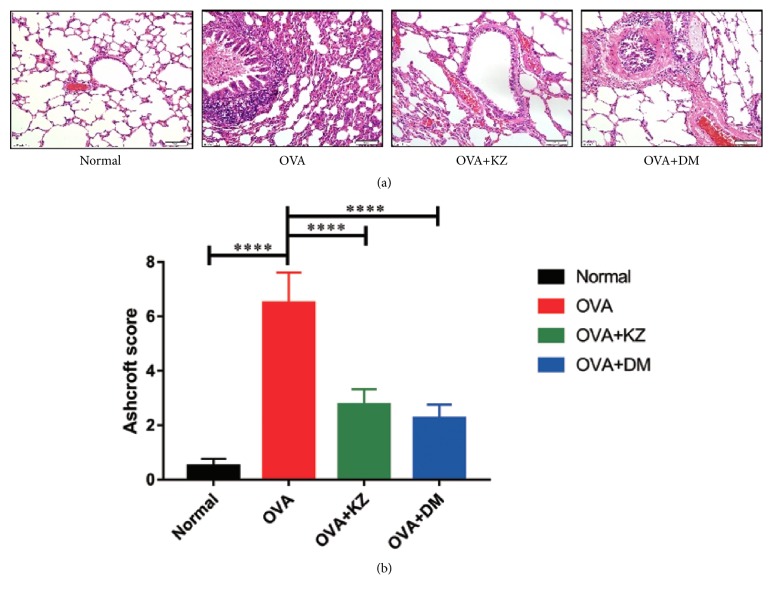
Morphological changes and the score of Ashcroft grading scale in each group. (a) Representative images of H&E staining in each group, indicating that Kangzhi syrup inhibited the inflammatory reaction in lung tissues. (b) On the basis of the Ashcroft grading scale for pulmonary fibrosis, Kangzhi syrup can significantly inhibit the fibrosis remodeling in CVA models by semiquantification under light microscope (10 × 40 magnification). The data are presented as the mean ± SEM. ^*∗∗∗∗*^*p* < 0.0001.

As shown in Figure 4, the hematoxylin-eosin staining was performed to observe the pathological changes in the groups. In the lung tissues of OVA group, we observed a marked infiltration of inflammatory cells into perivascular and peribronchial connective tissues compared with the lung tissues of normal group. There was no obvious collagen deposition around airway walls and blood vessels in the normal group, while it was markedly increased in the OVA group. In OVA-stressed CVA lung tissue, there was a marked infiltration of inflammatory cells into perivascular and peribronchial connective tissues. The treatments of Kangzhi syrup and Dexamethasone significantly reduced the infiltration of inflammatory cells and deposition of collagen in lung tissues. The further analysis of the pulmonary fibrosis revealed that the score of Ashcroft grading scale was significantly reduced in the OVA + KZ groups compared with OVA group (Figure 4).

### 3.5. Protein Content and mRNA Expression of TGF-*β*1 and NF-*κ*B in the Lung Tissue

**Figure 5:**
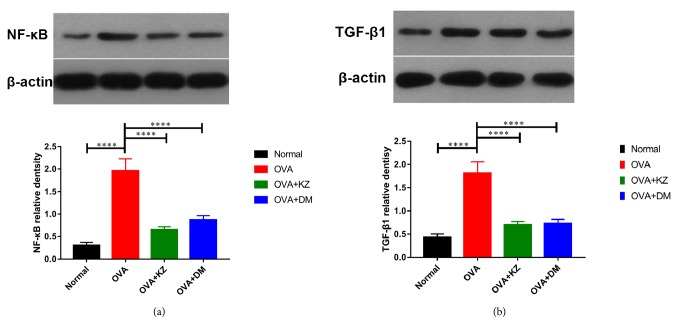
Effects of Kangzhi syrup on the expression of NF-*κ*B/P65 and TGF-*β*1 in lung tissues. (a-b) Representative Western blots of NF-*κ*B and TGF-*β*1 in each group. The data are presented as the mean ± SEM. ^*∗∗∗∗*^*p* < 0.0001.

To validate the anti-inflammatory activity of Kangzhi syrup in the lung tissue, the activation of TGF-*β*1 induced NF-*κ*B signaling pathway was evaluated by qRT-PCR and Western blotting (Figure 5). In accordance with the quantitative analysis of TGF-*β*1 in the BALF, OVA group showed a markedly enhanced TGF-*β*1 expression, and the treatments of Kangzhi syrup and Dexamethasone reduced the protein content and mRNA expression of TGF-*β*1 in lung tissues. Moreover, the downstream NF-*κ*B changed accordingly in the protein content and mRNA expression.

### 3.6. Immunohistochemistry Staining of TGF-*β*1 in Lung Sections

**Figure 6:**
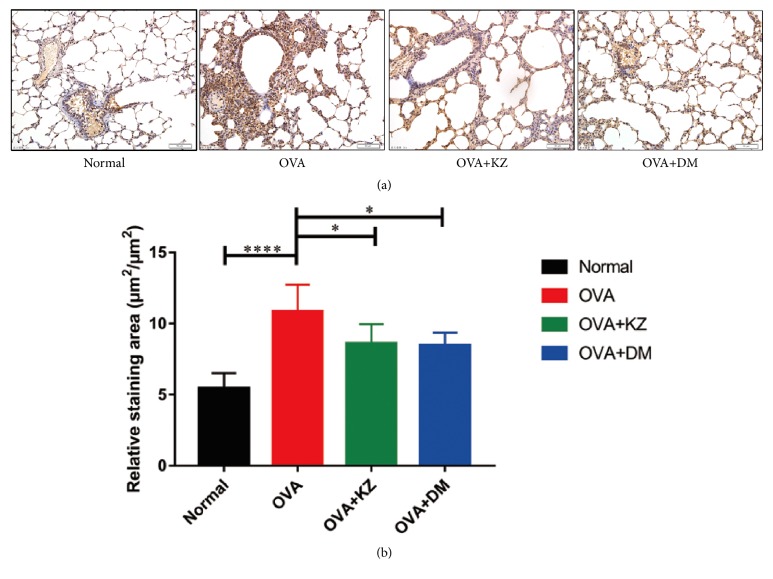
Semiquantitative analysis of IHC staining of TGF-*β*1. (a) Representative images of IHC staining of TGF-*β*1 in each group, confirming that Kangzhi syrup inhibited the expression of TGF-*β*1 in lung tissues by semiquantification under light microscope (10 × 40 magnification). The data are presented as the mean ± SEM. ^*∗*^*p* < 0.05; ^*∗∗∗∗*^*p* < 0.0001.

Similarly, TGF-*β*1 level in lung tissues was significantly increased by OVA challenge, which again was attenuated by Kangzhi syrup and Dexamethasone (Figure 6).

## 4. Discussion

As the air pollution has become more and more serious in China, the chronic cough is being considered a challenging problem in the area of respiratory medicine. Too strong and frequent cough severely deteriorates the quality of life of patients. Surveys revealed that CVA accounted for 24.0–33.3% of chronic cough cases [17, 18], which is commonly characterized by increased AHR and eosinophil accumulation. The main treatments for CVA include inhaled corticosteroid, bronchodilator, leukotriene receptor antagonist, and antibiotics [19]. Inhaled corticosteroid can inhibit the inflammation but cannot prevent the recurrence. Bronchodilators can relieve symptoms but cannot effectively reduce AHR. Prophylactic leukotriene receptor antagonist has some effect on asthma and CVA patients, but the effectiveness and side effects need further observation. Increasing evidence began to accumulate that TCM has shown significant efficacy in the treatment of CVA. However, the mechanism of treatment of TCM needs to be interpreted in some degree.

Kangzhi syrup was developed from a classic formula of TCM, which has long been used to treat chronic cough and CVA in our department of pediatric Chinese Medicine. The syrup preparation is a convenient manner and accessible to the pediatric patients. After a long-term clinical application, we have registered a qualification certificate in the local drug administrations. The commercial drug batch number was Z20131020. In this study, we aimed to clarify the underlying mechanism of Kangzhi syrup. According to the dose used in clinic, we converted the human dose to guinea pig dose using allometric scaling [20]. Our study clearly demonstrated that Kangzhi syrup had great potential on suppressing pulmonary inflammation, by which it could reduce the AHR and prevent airway remodeling in a guinea pig model of CVA. Moreover, the clinical dose of Dexamethasone was converted to a dose for guinea pigs and used as a positive control in the present study.

OVA-sensitized guinea pigs challenged with an aerosol of OVA have been used as animal models of airway allergic disease in many studies [14, 21–23]. The histological feature of this model was characterized by primarily eosinophilic pulmonary inflammation, but the AHR might occur in an OVA independent way [23]. A modified animal model of CVA, challenging those sensitized guinea pigs with inhalation of capsaicin, could enhance the AHR and the cough reflex sensitivity and was used as an animal model of CVA in this study [14]. Our data on inflammatory cells accumulation in BAL fluid in OVA-sensitized guinea pigs were in agreement with those published articles [14, 24]. Kangzhi syrup could significantly decrease the total number of leukocytes, which indicated the overall inflammatory reaction was suppressed by the treatment of Kangzhi syrup. Meanwhile, the percentage of eosinophil and lymphocytes in BAL fluid was also decreased, which explained why the Kangzhi syrup was peculiar to alleviate the syndromes of CVA. Concerning the increase of macrophages in the treatment of Kangzhi syrup and DM, that might also help to eliminate the neutrophils in order to reduce the inflammation in lung tissues. But overall, the anti-inflammatory effect of Kangzhi syrup definitely contributed to its antitussive effect. In addition to the decrease of absolute number of eosinophils, ECP level, as a basic protein released by activated eosinophils, was suppressed by Kangzhi syrup, which represents the active inflammatory reaction and significantly correlates with clinical severity in CVA [25, 26]. Moreover, there were differences with no significance in the antitussive effect of the Dexamethasone, suggesting that Kangzhi syrup could be used as a CAM of inhaled corticosteroid for treating CVA.

It is generally accepted that type 2 immune responses, stimulated by Th2 cells, are associated with allergy and asthma, which is characterized by high antibody titres and eosinophilia. TH2 cells mainly secrete the cytokines IL-4, IL-5, and IL-13 to mediate inflammatory and remodeling changes in the airway mucosa that predispose an individual to asthma and to asthma exacerbations. Compared with the other cytokines, IL-13 was reported to have critical roles in eosinophil induced airway AHR and mucus production [27, 28]. IL-13-directed treatment showed the efficacy in the maintenance of asthma control when corticosteroid dose was reduced in patients with moderate to severe asthma [29]. Effects are greatest in patients with high blood eosinophil levels. In our previous study, Kangzhi syrup could decrease IL-13 level in BALF of asthma models (data published) [30], which should contribute to the overall protective effects in the CVA model.

Patients with chronic coughs manifest marked changes in airway wall remodeling involving proliferation and differentiation of mesenchymal cells, particularly myofibroblasts and smooth muscle cells. TGF-*β*1 is one member of the family of structurally related growth factors whose expression is increased in the asthmatic airways and is a prime candidate contributing to airway remodeling in asthma. TGF-*β*1 can induce the expression of extracellular matrix components and stimulate the proliferation and hypertrophy of airway smooth muscle cells [31–33], suggesting a possible cause of increased thickness of airway tissues. In addition, TGF-*β*1 also has potential to contribute to the cellular inflammatory response by inducing mesenchymal cells and airway smooth muscle cells to release chemokines such as IL-8 and CCL2 [34–37]. On the other hand, TGF-*β*1 targeting treatments could improve asthmatic symptom [38, 39]. We demonstrated that Kangzhi syrup effectively decreased TGF-*β*1 concentration in BALF and attenuated its expression in the lung tissue induced by inhalation of capsaicin in OVA-sensitized guinea pigs. The suppression of collagen deposition was confirmed by the morphological study. Both Kangzhi syrup and DM decreased the collagen deposition around airway walls and blood vessels, while the number of infiltrating inflammatory cells and blood cells were also inhibited. Moreover, it was reported that the combination TGF-*β*1 and IL-13 synergistically upregulated eotaxin-1 that could markedly enhance tissue eosinophils [37]. Therefore, our data suggested that the Kangzhi syrup could attenuate the allergic TH2 response and eventually prevent lung dysfunction and airway remodeling.

It is now generally accepted that active T-cell immune responses in allergic respiratory disease are skewed toward the Th2 phenotype rather than Th1 immunity. The high IFN-*γ*-producing Th1 cells might contribute to skewing the immune response toward surviving Th2 cells [40]. On the other hand, the systemic inflammation with increased production of IL-1, IL-6, or TNF-*α* has an effect on the various processes in asthma, such as recruitment and adhesion of eosinophil to airway epithelial cells and bronchoconstriction [41]. Our data suggested that Th1-related cytokine, macrophage-derived IL-1*β*, was also significantly upregulated in the CVA model, and the downstream NF-*κ*B signaling pathway was also activated, which would dramatically stimulate the release of proinflammatory cytokines to worsen asthma. Kangzhi syrup might directly inhibit the Th1 cellular immune response and decrease the expression of transcript factor NF-*κ*B to attenuate the airway allergy.

Given the formula of Kangzhi syrup is a mixture of 15 herbs, effective targets of these herbal products in asthma therapy are also very complicated. Nevertheless,* Herba Ephedrae* in Kangzhi syrup has previously been proven to exert anti-inflammatory effects in various animal models or in vitro. For example,* Herba Ephedrae*-composed San'ao decoction has been shown to lower the percentage of eosinophil in peripheral blood and BALF and could attenuate IL-4-induced eotaxin expression [42]. In guinea pigs,* Herba Ephedrae* extract reduced the number of citric acid-induced laryngeal coughs [43]. However, the key principle of composition of TCM is based on the synergistic effects of medicinal herbs, and Kangzhi syrup should apparently transcend a single herb in asthma therapy.

In summary, the current study demonstrated that clinical commercial Kangzhi syrup could effectively attenuate the cough induced with the inhalation of capsaicin in OVA-sensitized guinea pigs and decrease the eosinophilic airway inflammation and airway remodeling, most likely through inhibition of type-2 immune response by downregulation of IL-13, INF-*γ*, and TGF-*β*1. In addition to the relevance with Th2 immunity, Kangzhi syrup showed the potential for the type-1 immune suppression by downregulation of IL-1. Our findings support a possible application of Kangzhi syrup as a therapeutic drug for patients with CVA.

## Figures and Tables

**Table 1:** 

Grade of fibrosis	Manifestations
0	Alveolar septa: no fibrotic burden at the most flimsy small fibers in some alveolar walls Lung structure: normal lung

1	Alveolar septa: isolated gentle fibrotic changes (septum ≤ 3 × thicker than normal) Lung structure: alveoli partly enlarged and rarefied, but no fibrotic masses present

2	Alveolar septa: clearly fibrotic changes (septum > 3 × thicker than normal) with knot-like formation but not connected to each other Lung structure: alveoli partly enlarged and rarefied, but no fibrotic masses

3	Alveolar septa: contiguous fibrotic walls (septum > 3 × thicker than normal) predominantly in whole microscopic field Lung structure: alveoli partly enlarged and rarefied, but no fibrotic masses

4	Alveolar septa: variable Lung structure: single fibrotic masses (≤10% of microscopic field)

5	Alveolar septa: variable Lung structure: confluent fibrotic masses (>10% and ≤50% of microscopic field). Lung structure severely damaged but still preserved

6	Alveolar septa: variable, mostly not existent Lung structure: large contiguous fibrotic masses (>50% of microscopic field). Lung architecture mostly not preserved

7	Alveolar septa: nonexistent Lung structure: alveoli nearly obliterated with fibrous masses but still up to five air bubbles

8	Alveolar septa: nonexistent Lung structure: microscopic field with complete obliteration with fibrotic masses

## Data Availability

Data will be made available to all interested researchers upon request.

## References

[1] Locksley R. M. (2010). Asthma and allergic inflammation. *Cell*.

[2] Grammer L. C., Greenberger P. A. (1992). Diagnosis and Classification of Asthma. *CHEST*.

[3] Fujimura M., Ogawa H., Nishizawa Y., Nishi K. (2003). Comparison of atopic cough with cough variant asthma: Is atopic cough a precursor of asthma?. *Thorax*.

[4] Nakajima T., Nishimura Y., Nishiuma T. (2005). Characteristics of patients with chronic cough who developed classic asthma during the course of cough variant asthma: A longitudinal study. *Respiration*.

[5] Lai K., Chen R., Lin J. (2013). A prospective, multicenter survey on causes of chronic cough in China. *CHEST*.

[6] Fahy J. V. (2015). Type 2 inflammation in asthma—present in most, absent in many. *Nature Reviews Immunology*.

[7] Ernst E. (1998). Complementary therapies for asthma: What patients use. *Journal of Asthma & Allergy Educators*.

[8] Arnold E., Clark C. E., Lasserson T. J., Wu T. (2008). Herbal interventions for chronic asthma in adults and children. *Cochrane Database of Systematic Reviews*.

[9] Clark C. E., Arnold E., Lasserson T. J., Wu T. (2010). Herbal interventions for chronic asthma in adults and children: a systematic review and meta-analysis. *Primary Care Respiratory Journal*.

[10] Park C. S., Kim T.-B., Lee J.-Y. (2012). Effects of add-on therapy with NDC-052, an extract from *Magnoliae flos*, in adult asthmatic patients receiving inhaled corticosteroids. *Korean Journal of Internal Medicine*.

[11] Chung W.-Y., Kim K. R., Jeong C.-K. (2010). Anti-inflammatory effects of licorice and roasted licorice extracts on TPA-induced acute inflammation and collagen-induced arthritis in mice. *Journal of Biomedicine and Biotechnology*.

[12] Li J., Zhao F. (2015). Anti-inflammatory functions of Houttuynia cordata Thunb. and its compounds: A perspective on its potential role in rheumatoid arthritis. *Experimental and Therapeutic Medicine*.

[13] Kim E. H., Shim B., Kang S. (2009). Anti-inflammatory effects of Scutellaria baicalensis extract via suppression of immune modulators and MAP kinase signaling molecules. *Journal of Ethnopharmacology*.

[14] Nishitsuji M., Fujimura M., Oribe Y., Nakao S. (2004). A guinea pig model for cough variant asthma and role of tachykinins. *Experimental Lung Research*.

[15] Hübner R.-H., Gitter W., El Mokhtari N. E. (2008). Standardized quantification of pulmonary fibrosis in histological samples. *BioTechniques*.

[16] Nishitsuji M., Fujimura M., Oribe Y., Nakao S. (2008). Effect of montelukast in a guinea pig model of cough variant asthma. *Pulmonary Pharmacology and Therapeutics*.

[17] Liu C. H., Shao M. J., Wang Q. (2013). Epidemiological survey of children asthma prevalence in Beijing urban area. *Zhonghua Yi Xue Za Zhi*.

[18] Respiratory Disease Group (P. C. o. S. M. A.) (2014). Epidemiological survey of asthma in children aged 0-14 years in seven districts of Shanghai. *Zhonghua er ke za zhi. Chinese journal of pediatrics*.

[19] Dicpinigaitis P. V. (2006). Chronic cough due to asthma: ACCP evidende-based clinical practice guidelines. *CHEST*.

[20] Nair A., Jacob S. (2016). A simple practice guide for dose conversion between animals and human. *Journal of Basic and Clinical Pharmacy*.

[21] Joachim R. A., Quarcoo D., Arck P. C., Herz U., Renz H., Klapp B. F. (2003). Stress enhances airway reactivity and airway inflammation in an animal model of allergic bronchial asthma. *Psychosomatic Medicine*.

[22] Muraki M., Tohda Y., Sugihara R., Nagasaka Y., Nakajima S. (1994). The Effect of TYB‐2285 on Dual Phase Bronchoconstriction and Airway Hypersensitivity in Guinea‐pigs Actively Sensitized with Ovalbumin. *Journal of Pharmacy and Pharmacology*.

[23] Sanjar S., Aoki S., Kristersson A., Smith D., Morley J. (1990). Antigen challenge induces pulmonary airway eosinophil accumulation and airway hyperreactivity in sensitized guinea-pigs: The effect of anti-asthma drugs. *British Journal of Pharmacology*.

[24] Jiao H.-Y., Su W.-W., Li P.-B. (2015). Therapeutic effects of naringin in a guinea pig model of ovalbumin-induced cough-variant asthma. *Pulmonary Pharmacology and Therapeutics*.

[25] D'Amato G., Liccardi G., Russo M., Saggese M., D'Amato M. (1996). Measurement of serum levels of eosinophil cationic protein to monitor patients with seasonal respiratory allergy induced by Parietaria pollen (treated and untreated with specific immunotherapy). *Allergy: European Journal of Allergy and Clinical Immunology*.

[26] Niimi A., Amitani R., Suzuki K., Tanaka E., Murayama T., Kuze F. (1998). Eosinophilic inflammation in cough variant asthma. *European Respiratory Journal*.

[27] Starkey M. R., Essilfie A. T., Horvat J. C. (2013). Constitutive production of IL-13 promotes early-life Chlamydia respiratory infection and allergic airway disease. *Mucosal Immunology*.

[28] Kuperman D. A., Huang X., Koth L. L. (2002). Direct effects of interleukin-13 on epithelial cells cause airway hyperreactivity and mucus overproduction in asthma. *Nature Medicine*.

[29] Wenzel S., Ford L., Pearlman D. (2013). Dupilumab in persistent asthma with elevated eosinophil levels. *The New England Journal of Medicine*.

[30] Li Z.-J., Liu Z.-W., Qu W.-Y. (2017). Effect of Kangzhi Syrup on IL-4,IL-13 and IFN-*γ* in BALF of Asthma Rats. *Acta Chinese Medicine & Pharmacology*.

[31] Vignola A., Chanez P., Chiappara G. (1997). Transforming Growth Factor- *β* Expression in Mucosal Biopsies in Asthma and Chronic Bronchitis. *American Journal of Respiratory and Critical Care Medicine*.

[32] Xie S., Sukkar M. B., Issa R. (2007). Mechanisms of induction of airway smooth muscle hyperplasia by transforming growth factor-beta. *American Journal of Physiology-Lung Cellular and Molecular Physiology*.

[33] Xie S., Macedo P., Hew M., Nassenstein C., Lee K., Chung K. F. (2009). Expression of transforming growth factor-*β* (TGF-*β*) in chronic idiopathic cough. *Respiratory Research*.

[34] Bossé Y., Stankova J., Rola-Pleszczynski M. (2010). Transforming growth factor-*β*1 in asthmatic airway smooth muscle enlargement: Is fibroblast growth factor-2 required?. *Clinical & Experimental Allergy*.

[35] Boxall C., Holgate S. T., Davies D. E. (2006). The contribution of transforming growth factor- and epidermal growth factor signalling to airway remodelling in chronic asthma. *European Respiratory Journal*.

[36] Jarai G., Sukkar M., Garrett S. (2004). Effects of interleukin-1*β*, interleukin-13 and transforming growth factor-*β* on gene expression in human airway smooth muscle using gene microarrays. *European Journal of Pharmacology*.

[37] Wenzel S. E., Trudeau J. B., Barnes S. (2002). TGF-beta and IL-13 synergistically increase eotaxin-1 production in human airway fibroblasts. *Journal of Immunology*.

[38] Ma C., Ma Z., Fu Q., Ma S. (2013). Curcumin attenuates allergic airway inflammation by regulation of CD4^+^CD25^+^ regulatory T cells (Tregs)/Th17 balance in ovalbumin-sensitized mice. *Fitoterapia*.

[39] Qin X., Zhang G., Zhang X. (2012). Protein tyrosine phosphatase SHP2 regulates TGF-*β*1 production in airway epithelia and asthmatic airway remodeling in mice. *Allergy*.

[40] Agrawal D. K., Shao Z. (2010). Pathogenesis of allergic airway inflammation. *Current Allergy and Asthma Reports*.

[41] Aslani M. R., Keyhanmanesh R., Khamaneh A. M. (2016). Lung Altered Expression of IL-1beta mRNA and Its Signaling Pathway Molecules in Obese-asthmatic Male Wistar Rats. *Iranian Journal of Allergy, Asthma and Immunology*.

[42] Yun L., Xin-Sheng F., Jing-Hua Y., Li X., Shan-Shan W. (2014). CD4+CD25+FOXP3+ T cells, Foxp3 gene and protein expression contribute to antiasthmatic effects of San'ao decoction in mice model of asthma. *Phytomedicine*.

[43] Minamizawa K., Goto H., Shimada Y., Terasawa K., Haji A. (2006). Effects of Eppikahangeto, a Kampo formula, and Ephedrae herba against citric acid-induced laryngeal cough in guinea pigs. *Journal of Pharmacological Sciences*.

